# Abnormal Uterine Bleeding: A Pictorial Review on Differential Diagnosis and Not-So-Common Cases of Interventional Radiology Management

**DOI:** 10.3390/diagnostics14080798

**Published:** 2024-04-11

**Authors:** Sara Viganò, Antonella Smedile, Caterina Cazzella, Paolo Marra, Pietro Andrea Bonaffini, Sandro Sironi

**Affiliations:** 1 Department of Radiology, Papa Giovanni XXIII Hospital, Piazza OMS 1, 24127 Bergamo, Italy; asmedile@asst-pg23.it (A.S.); ccazzella@asst-pg23.it (C.C.); pmarra@asst-pg23.it (P.M.); pa.bonaffini@gmail.com (P.A.B.); sandro.sironi@unimib.it (S.S.); 2 School of Medicine, University of Milano-Bicocca, Piazza dell’Ateneo Nuovo 1, 20126 Milano, Italy

**Keywords:** abnormal uterine bleeding, diagnostic radiology, interventional radiology, ultrasounds, computed tomography, magnetic resonance imaging

## Abstract

Abnormal uterine bleeding (AUB) poses a multifaceted challenge in women’s health, necessitating an integrated approach that addresses its diverse etiologies and clinical presentations. The International Federation of Gynecology and Obstetrics PALM-COEIN classification system provides a systematic approach to the diagnosis of AUB in non-pregnant women, based on clinical and imaging-based categorization of causes into structural (Polyps, Adenomyosis, Leiomyomas and Malignancy; PALM), and non-structural causes (Coagulopathies, Ovulatory disorders, primary Endometrial disorders, Iatrogenic and Not otherwise classified; COEIN). On the other hand, placental disorders, uterine rupture, ectopic pregnancy and retained products of conceptions are the main causes of uterine bleeding during pregnancy and in the peripartum period. Ultrasound is usually the first-line imaging technique for the differential diagnosis of causes of AUB. Computed Tomography may be useful if ultrasound findings are unclear, especially in emergency settings. Magnetic resonance imaging, when indicated, is an excellent second-line diagnostic tool for a better non-invasive characterization of the underlying cause of AUB. This pictorial review aims to illustrate the main causes of AUB from the point of view of diagnostic imaging and to show not-so-common cases that can be treated by means of interventional radiology.

## 1. Introduction

Abnormal uterine bleeding (AUB) in non-pregnant women of reproductive age is defined by the International Federation of Gynecology and Obstetrics (FIGO) as bleeding from the uterine corpus that is of abnormal duration, volume, frequency, and/or regularity. AUB may be chronic when it has been present for the previous 6 months, or acute when it is an episode of heavy bleeding that requires immediate intervention [1,2]. AUB is a common condition, with a prevalence estimated to be between 10% and 30% among women of reproductive age [3], and has an important impact on health-related quality of life and high economic costs for society [4]. The FIGO PALM-COEIN classification system, presented in 2011 and revised in 2018, provides a systematic approach to the diagnosis of AUB in women in reproductive age, based on clinical and imaging-based categorization of causes into structural (Polyps, Adenomyosis, Leiomyomas and Malignancy; PALM), and non-structural causes (Coagulopathies, Ovulatory disorders, primary Endometrial disorders, Iatrogenic and Not otherwise classified; COEIN) [5]. Postmenopausal abnormal uterine bleeding (PMB), defined as spontaneous uterine bleeding that occurs more than one year after the last menstrual period [6], is also a frequent condition occurring in 1–10% of women and accounting for 5% of referrals for gynecological examinations [7,8]. The prevalence of endometrial cancer in women having PMB is about 1–14%, while 90% of endometrial cancer patients present with PMB [4]. Therefore, the PALM-COEIN classification can be applied in cases of PMB, with a recommended initial diagnostic approach aimed at excluding the presence of endometrial malignancy or endometrial hyperplasia [9,10,11]. On the other hand, in pregnant women, abnormal uterine bleeding is a serious obstetrical condition, and uterine hemorrhage complicates 1–10% of all deliveries, being a leading cause of maternal mortality and morbidity [12]. Placental disorders, uterine rupture, ectopic pregnancy and retained products of conception are the main causes of uterine hemorrhage during pregnancy and in the postpartum period [13,14,15]. In patients presenting with AUB, the diagnosis is based on anamnesis, clinical and laboratory findings, and diagnostic imaging [3]. Transvaginal ultrasound (TVUS) is usually the first-line imaging technique for the evaluation of AUB both in premenopausal and in menopausal women [16]. Computed Tomography (CT) may be useful if ultrasound findings are equivocal, especially in emergency settings [17]. Magnetic resonance imaging (MRI), when indicated, is an excellent second-line diagnostic tool for a better non-invasive characterization of the underlying cause of AUB [18,19]. On this basis, this pictorial review aims (1) to summarize the range of conditions that may manifest with abnormal uterine bleeding in women in reproductive age, in post-menopausal women and during pregnancy or postpartum; (2) to describe the typical imaging findings, focusing on the structural causes of AUB that can be detected by different imaging techniques; and (3) to illustrate not-so-common cases of AUB that can be managed by an interventional radiology approach.

## 2. Relevant Sections

### 2.1. Differential Diagnosis

#### 2.1.1. Polyp: AUB-P

**Figure 1:**
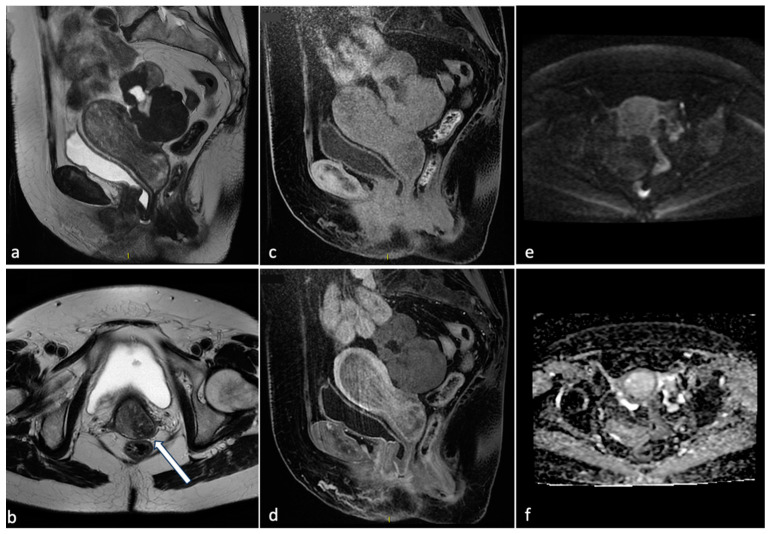
Voluminous endometrial polyp. A 76-year-old woman with post-menopausal bleeding. MRI shows on T2 sequences (**a**,**b**) a voluminous mass within the endometrial cavity of intermediate signal intensity with cystic components and a fibrous core (arrow). The mass demonstrates absence of endometrial invasion on pre- and post-contrast T1 sequences (**c**,**d**) and low signal on DWI with high signal on the ADC map (**e**,**f**). These findings are suggestive of partially expelled endometrial polyp.

Endometrial polyps are localized excess growth of endometrial glands and stroma that extend beyond the surface of the endometrial lining [20]. Endometrial polyps vary from a few millimeters to several centimeters in diameter and may have a broad base (sessile) or be attached by a pedicle (pedunculated) [21]. The prevalence of endometrial polyps is estimated to be from 10% to 30%, being lower in asymptomatic women of reproductive age and higher in women in postmenopausal age presenting with AUB [22]. Most endometrial polyps are benign. The risk of malignancy is related to postmenopausal status and to symptomatic AUB [23]. In a review and meta-analysis of the oncogenic potential of endometrial polyps, Uglietti et al. found that there was a significant difference in the prevalence of malignant polyps between postmenopausal and reproductive-age women, standing at 4.93% and 1.12% respectively. Furthermore, the prevalence of endometrial neoplasia within polyps was higher in women with symptomatic bleeding (5.14%) compared with those without bleeding (1.89%) [24]. The first-line imaging technique for the detection of endometrial polyps is TVUS, with a sensitivity range from 19% to 96% and a specificity between 53 and 100%; color-doppler, 3D TVUS and saline contrast sonohysterography improve the accuracy of 2D TVUS [25,26,27]. The typical ultrasound appearance of a benign endometrial polyp is a hyperechogenic endometrial focal lesion with or without regular small cysts. At Doppler ultrasound examination, an endometrial polyp is characterized by the presence of a single pedicle artery [28]. Several studies, however, show that these ultrasound signs have a lower sensitivity and specificity in women with postmenopausal bleeding and endometrial thickness equal or more than 5 mm [29]. Considering these limitations and the risk of malignancy, in selected cases, there could be an indication to perform MRI as a second-line diagnostic tool [30]. On MRI, endometrial polyps are typically of intermediate signal intensity (SI) compared to that of the endometrium on T2-weighted imaging (T2WI), show low SI on diffusion-weighted imaging (DWI), and show moderate contrast enhancement. Specific MRI findings are the presence of a central fibrous core (low SI on T2WI), intratumoral cysts (high SI on T2WI) and the absence of endometrial invasion [31,32] (Figure 1). According to guidelines, postmenopausal patients with AUB and a suspected endometrial polyp should undergo diagnostic hysteroscopy with polypectomy. Asymptomatic endometrial polyps in postmenopausal women and in young women should be removed in case of large diameter (>2 cm) [33].

#### 2.1.2. Adenomyosis: AUB-A

**Figure 2:**
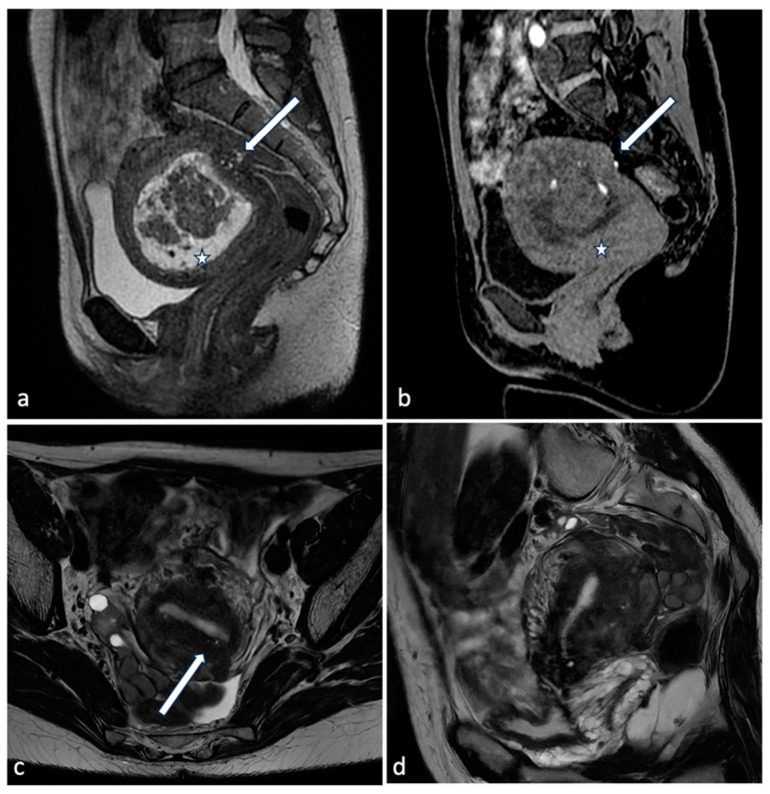
Different endometriosis phenotypes. A 41-year-old patient with a history of infertility, menometrorrhagias and chronic pelvic pain: MRI shows on sagittal CUBE T2WI (**a**, arrow) an extensive posterior external adenomyosis with bright foci on T1WI (**b**, arrow). There was associated deep pelvic infiltrating endometriosis and a large submucosal leiomyoma (**a**,**b**, star). Another 50-year-old patient with history of metrorrhagias: MRI demonstrates on axial T2WI (**c**) internal diffuse adenomyosis with hyperintense foci within the JZ (arrow). Sagittal T2 sequence (**d**) shows a diffuse thickening of the JZ (15 mm), prevalent on the posterior wall.

Adenomyosis is a benign gynecological disorder defined as the presence of ectopic endometrial glands and stroma within the myometrium, typically surrounded by hypertrophic and hyperplastic myometrium, often resulting in an enlarged uterine corpus [34]. Adenomyosis, even if most frequently asymptomatic, has been identified as one of the structural causes of AUB. The prevalence of adenomyosis is extremely variable, ranging from 20 to 80% according to age and to co-existing conditions such as endometriosis or infertility [35]. The exact etiology of adenomyosis remains unclear, but potential risk factors include hyper-estrogenism, endometriosis, pregnancy, and prior uterine surgeries [36]. The gold standard for the diagnosis is histological examination on a hysterectomy specimen, but advances in imaging modalities have made it possible to obtain accurate detection in cases of patients with a desire for conservative treatment [37,38]. TVUS and MRI are crucial for accurate diagnosis, with sensitivities of 72–78% and 77%, respectively, and specificities of 81–82% and 89%, respectively, with TVUS being operator dependent [39]. The prevailing recent perspective is that MRI may be the most accurate diagnostic tool for identifying the endometriosis phenotype [34]. MRI is also the preferred modality when there is association with other uterine pathologies, such as endometriosis and leiomyomas [40]. For TVUS, the MUSA (Morphological Uterus Sonographic Assessment) group proposed a consensus statement on terms and definitions to describe the sonographic features of adenomyosis [41]. Typical findings on TVUS include (a) asymmetrical thickening of the myometrial walls (b) myometrial cysts, (c) hyperechoic islands, (d) an irregular junctional zone (JZ) and (e) interrupted JZ. For MRI, Bazot et al. proposed a classification to distinguish between internal adenomyosis (subtypes: focal, diffuse, superficial), external adenomyosis (subtypes: posterior external, anterior external) and adenomyoma (subtypes: intramural cystic, intramural solid, subserosal; submucosal) [42]. Characteristics findings of internal adenomyosis on MRI are millimetric bright foci within the myometrium on T2WI, which can be hyperintense or isointense on T1WI according to the presence of cyclic hemorrhage, and an ill-defined endometrial junction. Indirect signs of internal adenomyosis are diffuse or localized thickening of the JZ considering a threshold thickness of 13 mm or a JZ to myometrium thickness ratio >40% [40]. On the other hand, external adenomyosis is defined by the presence of a subserosal mass on the posterior or anterior wall of the myometrium not affecting the JZ, which shows a hypointense signal on T2WI, with or without a hyperintense cystic component on T2WI and sometimes on T1WI. External adenomyosis is often associated with deep infiltrating pelvic endometriosis [43]. Adenomyomas appear as a mass within the myometrium, not affecting the serosa nor the JZ. Adenomyomas are usually hypointense on T2WI, with the possibility of hyperintense cystic foci on T2WI and/or hyperintense hemorrhagic foci on T2WI and on T1WI [44] (Figure 2). Management strategies for adenomyosis vary based on the severity of symptoms and the patient’s reproductive goals. Conservative approaches may involve analgesics, hormonal therapies, or intrauterine devices releasing progestins. In cases in which medical management proves inadequate or for those who are past childbearing age, more aggressive interventions such as uterine artery embolization or surgical options like hysterectomy may be considered [45].

#### 2.1.3. Leiomyoma: AUB-L

**Figure 3:**
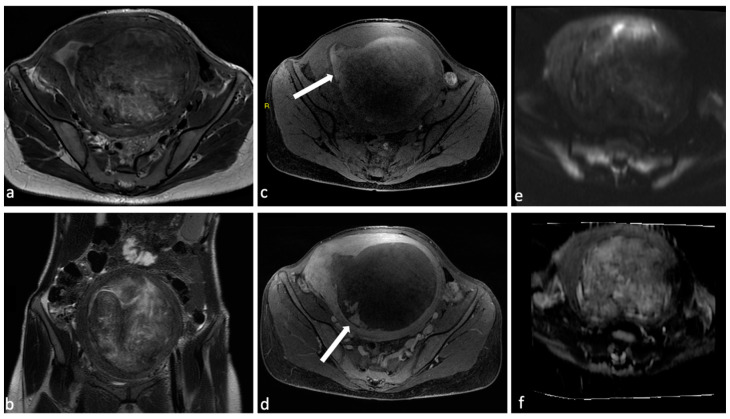
Leiomyoma with red degeneration. A 43-year-old woman with known submucosal myoma with recent onset of AUB and pelvic pain. On MRI, T2 axial (**a**) and coronal (**b**) sequences show enlargement of the known submucous leiomyoma with inhomogeneous signal intensity and peripheral rim hypointensity. The leiomyoma is characterized by signal hyperintensity on T1WI (**c**, arrow) and a small enhancing solid portion on post-contrast T1WI (**d**, arrow). There is no significant restricted diffusion/hypointensity on the ADC map (**e**,**f**).

Leiomyomas represent a common etiology of AUB among women of reproductive age. These benign smooth muscle tumors arising from the myometrium are the most common benign neoplasm of the uterus, with an estimated prevalence of 20–40% among women >30 years old, and a higher prevalence in black women [46]. Being estrogen-dependent, leiomyomas are rare before menarche. Most diminish in size after menopause and often enlarge during pregnancy or secondary to oral contraceptive therapy [47]. The FIGO classification system categorizes leiomyomas based on their location within the uterine wall into (a) submucosal (subtypes: pedunculated intracavitary, submucosal), (b) other (subtypes: intramural, subserosal, pedunculated subserosal, nonmyometrtrial location), and (c) hybrid, with both submucosal and subserosal components [48]. The first-line diagnostic imaging technique is TVUS, which allows for the visualization of the size, number and location of leiomyomas, with sensitivity and specificity ranging from 65% to 99%, varying with the experience of the operator [49,50]. Classically, on TVUS, typical leiomyomas appear as solid, well-defined, homogeneous hypoechoic masses that may show posterior acoustic shadowing. However, due to the frequent differences in the composition of the extracellular matrix and fibroblasts or due to the presence of calcification and necrosis, leiomyomas may have different echogenicity [51]. MRI provides greater detail, aiding in the assessment of leiomyomas’ characteristics, location, and their impact on adjacent structures, which is particularly valuable in treatment planning. On MRI, typical uterine leiomyomas appear as well-circumscribed lesions, with low signaling on T2WI and T1WI and on both diffusion-weighted imaging (DWI) and apparent diffusion coefficient (ADC) maps, with the so-called “blackout effect” due to the fibrous component. On post-contrast imaging, they show variable enhancement patterns [52]. MRI may be useful in the characterization of atypical myomas with different imaging features such as edema, degeneration (cystic, myxoid, red degeneration), and different histologic subtypes [53]. Myomas with cystic degeneration contain fluid areas, appearing with low signal intensity on T1WI and high signal intensity on T2WI, without contrast enhancement [54]. A myoma with myxoid degeneration typically presents as a mass within the myometrium, with heterogeneous components that tend to exhibit lower signal intensity than muscle on T1WI and higher peripheral signal intensity (due to myxoid deposition) on T2WI. On DWI, the tumor does not show restricted diffusion due to its myxoid composition, which aligns with the heterogeneous hyperintensity observed on T2WI. Leiomyomas with red degeneration typically appear hyperintense on T1WI and exhibit inhomogeneous hypointensity with a rim of low signal intensity on T2WI. The extent of contrast enhancement varies depending on the degree of infarction. In DWI, inhomogeneous restricted diffusion is observable, with a surrounding hypointense margin on the ADC map [55] (Figure 3). Cellular leiomyomas (CL), on the other hand, may show global or focal signal hyperintensity on T2WI and lower ADC values compared to degenerated leiomyomas but higher values compared to uterine sarcomas (US). Wang et al. found a cutoff ADC value of 1239 × 10^−6^ mm^2^/s for differentiating CL from degenerated leiomyomas, and a cutoff ADC value of 839 × 10^−6^ mm^2^/s for differentiating CL from US [56].

**Figure 4:**
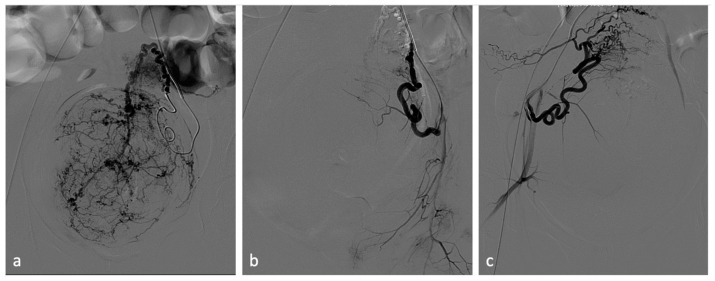
Uterine artery embolization for leiomyoma. A 27-year-old patient presenting with menometrorrhagias and pelvic pain. TVUS and MRI demonstrated a voluminous isthmic uterine leiomyoma (12 cm of diameter). Selective left uterine artery arteriogram (**a**) shows multiple vessels feeding the large leiomyoma. Embolization through the release of embolizing microparticles was performed. Post-embolization left artery arteriogram (**b**) shows good devascularization of the leiomyoma. Post-embolization selective right uterine arteriogram (**c**) showed ectasia of the right tubal artery with origin of tubo-ovarian trunk for the ipsilateral annexal branches. Given the high risk of non-target embolization, embolization of the right uterine artery was not performed.

The management of leiomyomas is multifaceted and depends on factors such as the severity of symptoms, the size and location, and the patient’s reproductive goals. For those seeking fertility preservation, myomectomy, either through hysteroscopy or laparoscopy, may be considered. Uterine artery embolization presents a non-surgical alternative [57] (Figure 4). In more severe cases or for women who have completed childbearing, hysterectomy remains a definitive solution [58,59].

#### 2.1.4. Malignant and Premalignant: AUB-M

**Figure 5:**
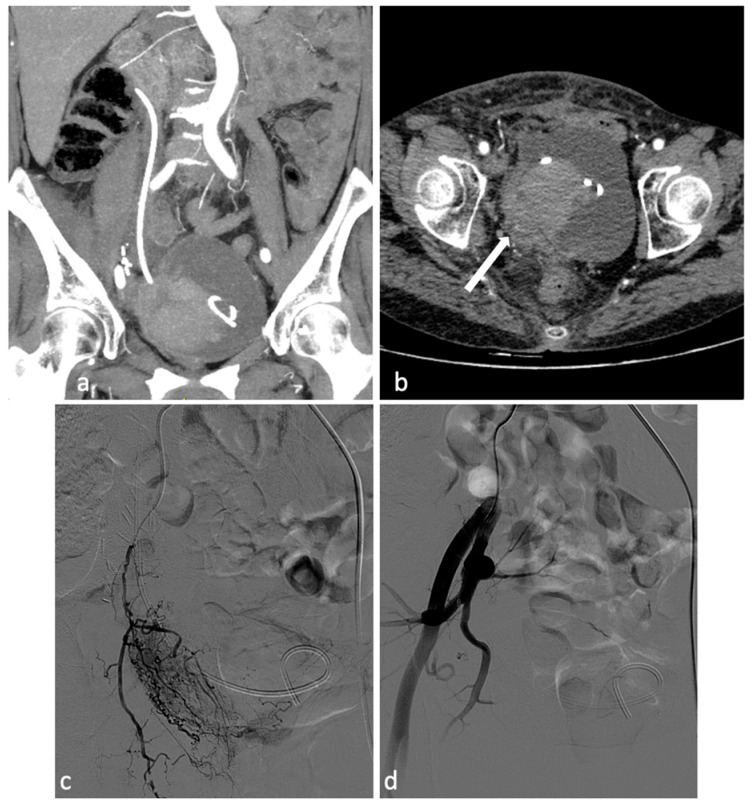
Pelvic recurrence of endometrial neoplasm causing vaginal bleeding. A 65-year-old patient with endometrial neoplasm treated by total hysterectomy, salpingo-oophorectomy, and right uretero-neocystostomy with DJ catheter placement, presenting with significant vaginal bleeding. CT investigation (**a**,**b**) shows a neoplastic mass (4 × 3 cm) in the right pelvis involving the bladder trigone, distal ureter, right bladder dome and vaginal dome. Concomitant intravesical clot measuring at least 6 cm and a clot in the right vaginal dome (**b**, arrow). Selective catheterization of the right hypogastric artery was performed. Angiography confirmed the presence of a hypervascular pathological formation of the right pelvis, with disorganized arterial hypervascularization (**c**). After superselective coaxial microcatheterization of the main afferent branch of the lesion, its embolization with PVA particles (350–500 microns) and occlusion with a 2 × 25 mm metal microspiral was performed. Control angiography shows effective devascularization of the lesion (**d**).

AUB-M refers to women with AUB and associated malignant and premalignant lesions of the uterus (e.g., endometrial carcinoma, leiomyosarcoma, and atypical endometrial hyperplasia). The risk of malignant and premalignant lesions of the uterus rises with age and with post-menopausal status. Although the literature indicates that endometrial atrophy is the main cause of postmenopausal bleeding (PMB), it must be considered that 1–10% of women with PMB are diagnosed with endometrial carcinoma (EC). In addition, AUB and PMB are the initial symptoms in 75% to 90% of patients with EC [8]. The first-line imaging technique for the detection of malignancies is TVUS, which allows for detailed visualization of the endometrium and myometrium, aiding in the assessment of thickness, echogenicity, and vascular patterns. According to the literature, an endometrial thickness of 4 mm or less on TVUS has a greater than 99% negative predictive value for EC [60]. Using the International Endometrial Tumor Analysis (IETA) terminology, Van Den Bosch et al. described the typical ultrasound features of endometrial pathologies [61]. Once endometrial hyperplasia or cancer is suspected, endometrial sampling is imperative [10]. An endometrial biopsy provides the definitive diagnosis of EC with the definition of histological subtypes, grade, depth of invasion and the presence/absence of lymphovascular space invasion. According to the ESGO guidelines, molecular classification is encouraged in all endometrial cancer [62]. Histopathological and molecular classification help stratify patients based on the definition of prognostic risk groups (low, intermediate, high-intermediate, and high and advanced) [62]. This stratification also enables the identification of distinct therapeutic strategies with the future prospect of treatments tailored to individual patient profiles [63]. EC is commonly staged according to the FIGO staging system, and surgical staging is the standard of care (total hysterectomy, salpingo-oophorectomy, peritoneal washing, and lymph node assessment) [64]. Preoperative imaging for the assessment of local extent is crucial for treatment planning. MRI is the preferred second-line diagnostic tool for the assessment of local extent concerning myometrial invasion (<50% or >50%), involvement of adjacent structures and pelvic metastasis. On MRI, EC is isointense to adjacent normal endometrium on T1WI, while it shows an intermediate heterogeneous signal on T2WI. Sagittal and axial oblique (perpendicular to the endometrial cavity) 2D T2W sequences are mandatory to stage endometrial cancer. Functional MRI sequences are crucial for the assessment of myometrial invasion (MI). Dynamic contrast-enhanced imaging (DCE-MRI) allows the detection of the presence of uninterrupted enhancement of the subendometrial zone at 35–40 s after contrast administration [62], and the optimal timing for the evaluation of MI is 2 min 30 s (equilibrium phase) [65]. Delayed phase (3–4 min) is useful for the assessment of cervical invasion. On DWI, EC shows a high signal intensity with low signal intensity on the ADC map [66,67]. On this basis, in 2019, the updated guidelines of the European Society of Urogenital Radiology (ESUR) recommended the use of DCE-MRI or a single post-contrast phase at 2 min 30 s and DWI (with a minimum of two b values of 0 and 800–1000 s/mm^2^ and at least one plane, preferably an axial oblique plane perpendicular to the endometrial cavity). Functional sequences are also useful in challenging cases, e.g., large tumor, tumor isointense to myometrium on T2WI, coexisting leiomyomas or adenomyosis [62]. Gynecological malignancies may also present with vaginal bleeding in cases of advanced stages, with the invasion of adjacent structures such as the bladder and vaginal fornix. In these cases, when surgical treatment is not an option due to a high risk of complication, interventional arterial embolization is the treatment of choice to interrupt the bleeding and repristinate blood parameters and hemodynamic stability [68] (Figure 5).

#### 2.1.5. Ectopic Pregnancy

**Figure 6:**
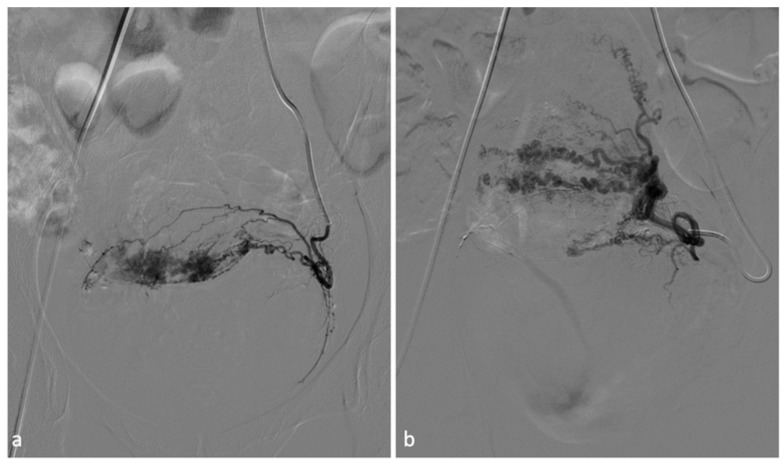
Scar pregnancy. A 25-year-old female patient with pregnancy on a previous caesarean section scar, considered at high risk. It was decided to perform a uterine embolization procedure prior to curettage. After selective catheterization of the uterine arteries (**a**), bilateral embolization with gelfoam and calibrated particles was performed. Post-procedural angiography (**b**) demonstrated satisfactory devascularization of scar pregnancy.

Ectopic pregnancy (EP), defined as the implantation of a fertilized egg outside the uterine cavity, poses a significant and potentially life-threatening cause of uterine bleeding, being the leading cause of maternal mortality in the first trimester, with an incidence of 5–10% of all pregnancy-related deaths [69]. The prevalence of EP has seen a gradual rise in recent years, attributable in part to increased rates of pelvic inflammatory disease, tubal damage, and assisted reproductive technologies [70]. While ectopic pregnancies can occur in various locations, the fallopian tube is the most common site, accounting for approximately 95% of cases. Other usual locations are in the cervix (1%), in the ovaries (0.5–3%) and along a previous cesarian scar (6%) [71] (Figure 6).

**Figure 7:**
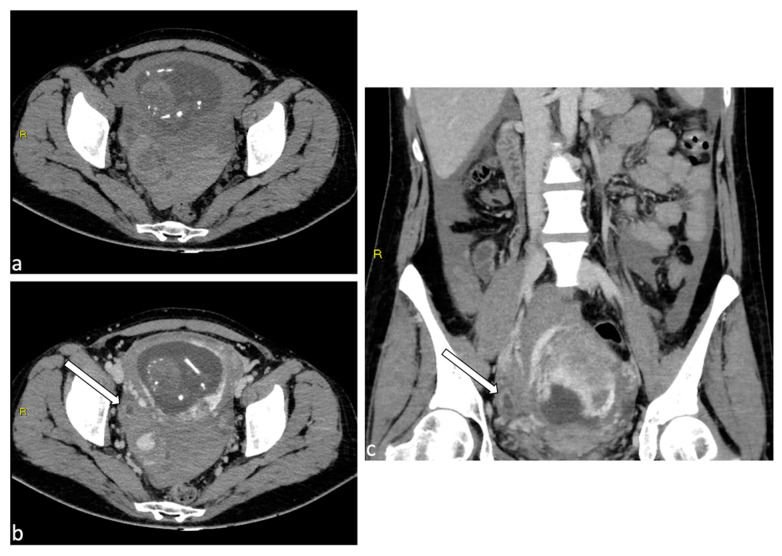
Heterotopic pregnancy. A 35-year-old patient pregnant at 17 weeks with sudden abdominal pain, vaginal bleeding, and hypotension. Transabdominal ultrasound documented viable intrauterine fetus and hemoperitoneum. Given the unavailability of MRI, an urgent CT scan was performed, which showed abundant hemoperitoneum (**a**), intact gravid uterus and a cystic mass with peripheral enhancement within the right fallopian tube (**b**,**c**, arrows). An emergency laparoscopy was performed and revealed a congested and swollen right salpinx, site of an ectopic pregnancy. A right salpingectomy was performed. The patient was then discharged and successfully completed the intrauterine pregnancy.

Timely diagnosis is crucial to prevent complications such as rupture and hemorrhage. EP rupture is marked by symptoms such as severe abdominal pain, vaginal bleeding with the possibility of massive intra-abdominal bleeding and even hemorrhagic shock [72]. After quantitative measurement of serum beta-human chorionic gonadotropin (β-hCG) levels, both transabdominal US and TVUS are crucial for the diagnosis. The typical US findings are an empty uterine cavity with a decidualized endometrium. A typical sign is the “tubal ring sign”, a ring-shaped lesion with an echoic center and a hyperechoic, hypervascular rim, representing the gestational sac. Free peritoneal fluid or hemoperitoneum may be observed [73]. If the US findings are inconclusive, MRI may be helpful in providing a more accurate anatomic localization. Key MRI findings include a gestational sac-like structure with low SI on T1WI and high SI on T2WI, dilatated tuba due to hemosalpinx, and tubal wall enhancement. Hemoperitoneum (free fluid of high SI on T1WI) may be present as an indirect sign of a ruptured EP [74]. US and MRI are the primary imaging modalities for evaluating gynecologic pathology; however, CT is frequently performed as the initial imaging modality in the setting of trauma or in emergency settings when hemoperitoneum of unknown origin is suspected and MRI is not available. CT usually shows a pelvic cystic mass with variable peripheral enhancement, with or without hemoperitoneum [75] (Figure 7). Treatment modalities for ectopic pregnancy depend on factors such as gestational age, hemodynamic stability, and the desire for future fertility. Medical management with methotrexate is often suitable for stable patients with unruptured ectopic pregnancies. Surgical intervention remains the preferred option for cases requiring immediate intervention [76].

#### 2.1.6. Uterine Rupture

**Figure 8:**
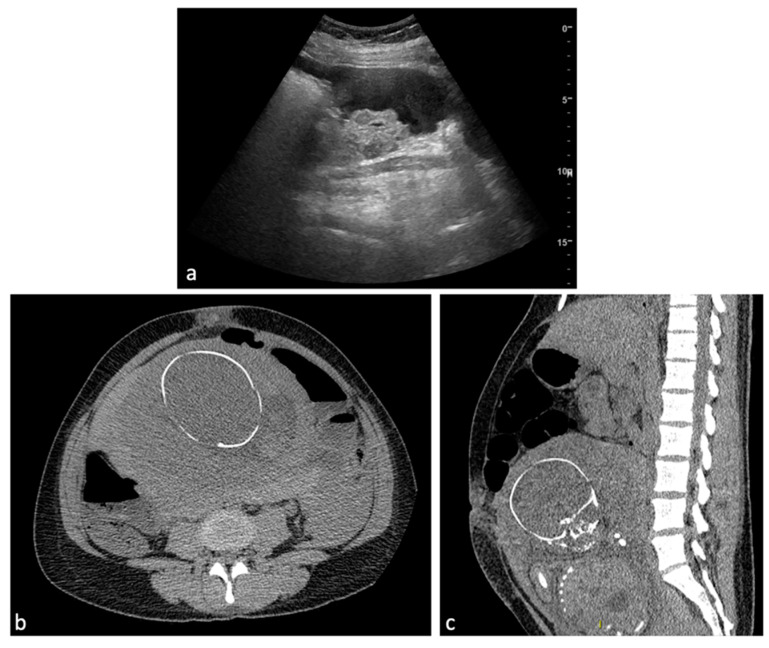
Uterine rupture. A 39-year-old female patient with a history of laparoscopic myomectomy who was pregnant at 29 weeks presented to the emergency department with abdominal pain and vaginal bleeding. Transabdominal ultrasound demonstrated hemoperitoneum (**a**) and a live fetus. CT scan without contrast medium (axial, **b**; sagittal, **c**) was performed urgently with confirmation of abundant hemoperitoneum. An urgent caesarean section was performed, with evidence of uterine rupture at the posterior wall in the presence of placenta accreta. Hysterectomy was performed.

Uterine rupture is a rare and potentially fatal obstetric complication defined as a full thickness tear of the uterine wall, typically occurring during labor or delivery. The most common etiological factor is a previous uterine scar, often resulting from a previous cesarean section [77]. Other risk factors include a history of uterine surgery, trauma, or congenital uterine anomalies. Uterine rupture may manifest as sudden and severe abdominal pain, vaginal bleeding and hemoperitoneum rapidly progressive to maternal shock. US in the initial evaluation may show an abnormality in the uterine wall, a hematoma next to a hysterotomy scar, free fluid in the peritoneum, or fetal parts outside the uterus [78] (Figure 8). Uterine rupture necessitates immediate surgical intervention, often requiring an emergency cesarean section.

#### 2.1.7. Placental Disorders

**Figure 9:**
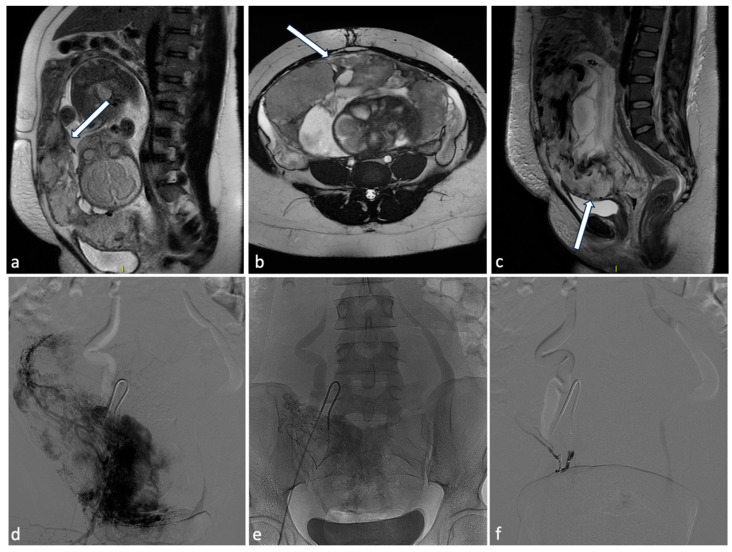
Placenta percreta. A 38-year-old woman at 27 weeks of gestation with placenta previa percreta. Sagittal (**a**) and axial (**b**) T2W SSFSE images show a heterogeneous placental signal with some low signal intensity bands representing venous lakes (arrows). There is also a focal uterine bulge with thinning of the overlying myometrium. The patient underwent a caesarean section and extraction of the fetus but no secondment due to placenta percreta. In the post-partum MRI (**c**), signs of placental percreta were still present with an image of bladder infiltration (arrow), later confirmed at cystoscopy. It was decided to perform endovascular devascularization of the placenta percreta left in situ before proceeding to hysterectomy. Preliminary arteriography of the abdominal aorta documented hypertrophy of both uterine arteries, clearly greater on the right, which exuberantly vascularized the hypertrophied placenta, supplying multiple intraplacental high-flow vascular lakes (**d**). After superselective catheterization with a coaxial microcatheter of both uterine arteries and some of their distal branches, bilateral embolization was performed by infusion of a suspension of embolizing particles. The procedure was completed with release of 3 platinum microspirals (3 mm diameter) at the level of the distal right uterine artery (**e**), proximal to the origin of the main collaterals and 2 microspirals (3 mm) at the origin of the left uterine artery. At the end of the operation, satisfactory uterine and placental devascularization was shown (**f**).

Placenta Accreta Spectrum (PAS) disorders encompass a spectrum of pathological conditions characterized by abnormal placentation, leading to potential complications, from vaginal bleeding to massive obstetrical hemorrhage [14,16]. These disorders, which include placenta accreta, increta, and percreta, arise when the placental villi invade and adhere to the myometrium beyond the normal depth, often reaching the uterine serosa or adjacent organs [79]. US is the first-line technique for the evaluation of the placenta. However, when US findings are equivocal, MRI is the second-line diagnostic tool to evaluate PAS disorders. Dark intraplacental bands on T2WI, increased placental thickness, placental/uterine bulge, myometrial thinning, bladder wall interruption, focal exophytic mass, and abnormal vascularization of the placental bed are the main MRI findings suggestive of a PAS disorder [80,81,82] (Figure 9). Antenatal recognition of PAS disorders is crucial for optimizing management strategies, which may include planned cesarean delivery, preoperative endovascular interventions, and coordination with a multidisciplinary team to mitigate the risk of severe bleeding and associated maternal morbidity [57,83]. 

#### 2.1.8. Retained Products of Conception

**Figure 10:**
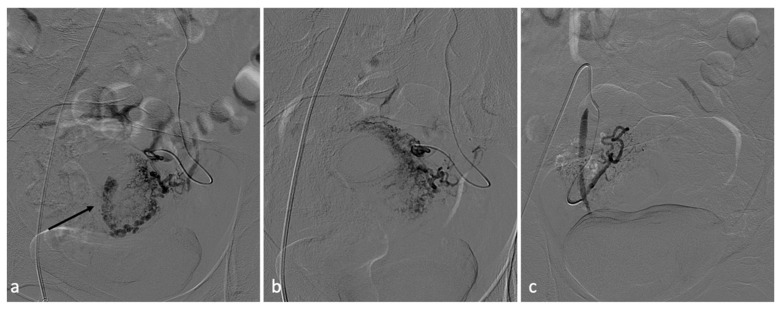
RPOC. A 25-year-old patient who continued to experience vaginal bleeding 1 month after natural birth. Ultrasound performed at another center howed the presence of placental residue suspicious for accreted succenturiate cotyledon. It was, therefore, decided to perform an embolization procedure. Bilateral hypogastric angiography documented the presence of hypervascular formation in the central portions of the uterus (**a**). After superselective coaxial microcatheterization of the uterine arteries, they were embolized with absorbable material (**b**). Control angiography documented effective vascularization of the lesion (**c**).

Retained products of conception (RPOC) typically occur after medical abortion after the first trimester or can manifest after either vaginal or cesarean delivery. This condition often manifests with symptoms such as AUB, lower abdominal and pelvic pain, and vaginal discharge due to infection. It can also cause late complications like the development of intrauterine adhesions and subfertility [84,85]. The diagnosis of RPOC is commonly supported by ultrasonography, with color Doppler imaging, which can help detect an associated underlying AVM. Typical TVUS findings include a thickened endometrium with a hyperechoic endometrial solid mass with prominent low-resistance color Doppler flow [86,87]. Traditionally, the management of RPOC involved blind dilation and suction curettage (D and C). However, contemporary approaches include expectant management, uterine artery embolization, and hysteroscopic resection of RPOC, all of which have proven to be safe and efficient alternatives [88] (Figure 10).

## 3. Conclusions

Many gynecological and obstetrical pathologies may manifest with abnormal uterine bleeding. Diagnostic imaging is necessary for identifying the underling etiology of AUB and discriminating conditions posing potential immediate risk to life and those with oncogenic potential from those that can be managed conservatively. Radiologists must possess a thorough understanding of the prevalent imaging findings, detected through US, CT, and MRI, in order to ensure prompt and accurate diagnosis, thereby enabling effective collaboration with clinicians.

## Data Availability

The data presented in this study are available on request from the corresponding author.
